# Tuberculosis treatment outcomes among disadvantaged patients in India

**DOI:** 10.5588/pha.16.0107

**Published:** 2017-06-21

**Authors:** C. Jackson, H. R. Stagg, A. Doshi, D. Pan, A. Sinha, R. Batra, S. Batra, I. Abubakar, M. Lipman

**Affiliations:** 1 Institute for Global Health, University College London (UCL), London, UK; 2 Operation ASHA, New Delhi, India; 3 Medical School, Imperial College London, London, UK; 4 Public Health England, London, UK; 5 UCL Respiratory, Division of Medicine, UCL, London, UK; 6 Royal Free London National Health Service Foundation Trust, London, UK

**Keywords:** epidemiology, directly observed therapy, slums, mycobacteria

## Abstract

**Setting:** Urban slums and poor rural areas in India, 2012–2014.

**Objective:** To describe the characteristics of tuberculosis (TB) patients enrolled in treatment through Operation ASHA, a non-governmental organisation serving disadvantaged populations in India, and to identify risk factors for unfavourable treatment outcomes.

**Design:** This was a retrospective cohort study. Patient characteristics were assessed for their relationship with treatment outcomes using mixed effects logistic regression, adjusting for clustering by treatment centre and Indian state. Outcomes were considered favourable (cured/treatment completed) or unfavourable (treatment failure, loss to follow-up, death, switch to multidrug-resistant TB treatment, transfer out).

**Results:** Of 8415 patients, 7148 (84.9%) had a favourable outcome. On multivariable analysis, unfavourable outcomes were more common among men (OR 1.31, 95%CI 1.15–1.51), older patients (OR 1.12, 95%CI 1.04–1.21) and previously treated patients (OR 2.05, 95%CI 1.79–2.36). Compared to pulmonary smear-negative patients, those with extra-pulmonary disease were less likely to have unfavourable outcomes (OR 0.72, 95%CI 0.60–0.87), while smear-positive pulmonary patients were more likely to have unfavourable outcomes (OR 1.38, 95%CI 1.15–1.66 for low [scanty/1+] and OR 1.71, 95%CI 1.44–2.04 for high [2+/3+] positive smears).

**Conclusion:** The treatment success rate within Operation ASHA is comparable to that reported nationally for India. Men, older patients, retreatment cases and smear-positive pulmonary TB patients may need additional interventions to ensure a favourable outcome.

## Abstract (FR)

**Contexte:** Bidonvilles urbains et zones rurales pauvres, Inde, 2012–2014.

**Objectif:** Décrire les caractéristiques des patients atteints de tuberculose (TB) enrôlés dans un traitement à travers l'Opération ASHA, une organisation non-gouvernementale au service des populations désavantagées en Inde, et identifier les facteurs de risque de résultat défavorable du traitement.

**Schéma:** Etude rétrospective de cohorte. Les caractéristiques des patients ont été évaluées en fonction de leur relation avec les résultats du traitement grâce à une régression logistique à effets mixtes ajustée sur le regroupement par centre de traitement et par l'état d'Inde. Le résultat a été considéré comme favorable (guéri/traitement achevé) ou défavorable (échec du traitement, perte de vue, décès, évolution vers un traitement de TB multirésistante, transfert).

**Résultats:** De 8415 patients, 7148 (84,9%) ont eu un résultat favorable. En analyse multi-variable, les résultats défavorables ont été plus fréquents parmi les hommes (OR 1,31 ; IC 95% 1,15–1,51), les patients plus âgés (OR 1,12 ; IC95% 1,04–1,21) et les patients déjà traités (OR 2,05 ; IC95% 1,79–2,36). Comparés aux patients atteints de TB pulmonaire à frottis négatif, les patients atteints de TB extra-pulmonaire ont été moins susceptibles d'avoir un résultat défavorable (OR 0,72 ; IC95% 0,60–0,87), tandis que les patients atteints de TB pulmonaire à frottis positif ont été plus susceptibles d'avoir un résultat défavorable (OR 1,38 ; IC95% 1,15–1,66 pour les frottis positifs faibles [rares/1+] et OR 1,71 ; IC95% 1,44–2,04 pour les frottis élevés [2+/3+]).

**Conclusion:** Le taux de succès du traitement dans le cadre de l'Opération ASHA est comparable à celui rapporté au niveau national en Inde. Les hommes, les patients plus âgés, les cas en retraitement et les patients atteints de TB pulmonaire à frottis positif pourraient avoir besoin d'interventions supplémentaires afin d'assurer un résultat favorable.

## Abstract (ES)

**Marco de referencia:** Barriadas urbanas y zonas rurales pobres en la India del 2012 al 2014.

**Objetivo:** Describir las características de los pacientes con tuberculosis (TB) inscritos en tratamiento en el marco de la Operación ASHA, que es una organización no gubernamental que atiende a las poblaciones desfavorecidas en la India, y determinar los factores de riesgo de obtener desenlaces terapéuticos desfavorables.

**Método:** Un estudio retrospectivo de cohortes. Las características de los pacientes se evaluaron con respecto a los desenlaces terapéuticos, mediante un análisis de regresión logística de efectos mixtos y ajuste con relación a los conglomerados, según el centro de tratamiento y el estado de la India. Los desenlaces se consideraron favorables (curación o tratamiento completo) o desfavorables (fracaso terapéutico, pérdida durante el seguimiento, muerte, cambio de tratamiento por TB multirresistente o transferencia a otro centro).

**Resultados:** De los 8415 pacientes, en 7148 el desenlace fue favorable (84,9%). El análisis multivariante reveló que los desenlaces desfavorables eran más frecuentes en los hombres (OR 1,31; IC95% 1,15–1,51), los ancianos (OR 1,12; IC95% 1,04–1,21) y en los pacientes con antecedente de tratamiento antituberculoso (OR 2,05; IC95% 1,79–2,36). Tomando como referencia a los pacientes con baciloscopia negativa, los desenlaces desfavorables fueron menos probables en los pacientes con TB extrapulmonar (OR 0,72; IC95% 0,60–0,87) y más probables en los pacientes con TB pulmonar y baciloscopia positiva (OR 1,38; IC95% 1,15–1,66 para las baciloscopias bajas, de escasos bacilos a 1+ y OR 1,71; IC95% 1,44–2,04 para las baciloscopia altas, de 2+ o 3+).

**Conclusión:** La tasa de éxito del tratamiento antituberculoso en el marco de la Operación ASHA es equivalente a la notificada a escala nacional en la India. Los pacientes de sexo masculino, los ancianos, los casos en retratamiento y los que presentan una TB pulmonar con baciloscopia positiva pueden necesitar intervenciones complementarias a fin de fomentar los desenlaces favorables.

Despite recent declines in reported tuberculosis (TB) rates in India, the disease remains a major public health challenge.^1^,^2^ With an estimated 2.8 million incident cases in 2015 (217 per 100 000 population),^1^ India is considered a high-burden country for TB by the World Health Organization (WHO). The reported treatment success rate was 88% for new and relapse cases registered in 2013^3^ and 74% for those registered in 2014;^1^ the apparent decline is most likely influenced by revisions to the estimates of the overall TB burden for the period 2000–2015.^1^

In India, most health care is delivered through the private sector, where >70% of health care contacts^4^,^5^ and an estimated 60% of TB patients^6^ are seen. Out-of-pocket expenditure can be high^5^ and the poorest individuals often have limited health care access.^7^ Socio-economic position (SEP) is associated with an increased risk for TB,^8^ delays in seeking treatment^9^ and loss to follow-up (LTFU).^10^ Other risk factors for TB disease include male sex, previous anti-tuberculosis treatment, alcohol consumption, increasing age, low body mass index (BMI) and tobacco use;^8^ several of these factors are also associated with LTFU and death during treatment.^10^,^11^

In this article, we identify risk factors for unfavourable treatment outcomes in a vulnerable population receiving treatment through the non-governmental organisation (NGO) Operation ASHA (New Delhi, India), which aims to enhance case identification and treatment delivery for patients who might not seek care through the Revised National Tuberculosis Control Programme (RNTCP).

## STUDY POPULATION AND METHODS

### Data collection

**FIGURE 1:**
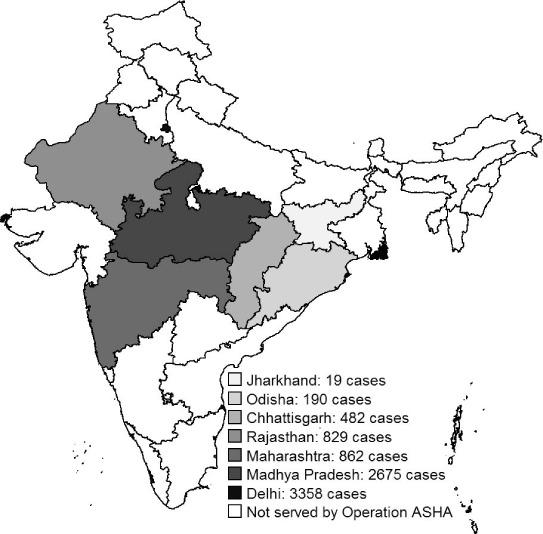
Geographical distribution of tuberculosis patients treated through Operation ASHA across seven Indian states.

Operation ASHA (New Delhi, India, www/opasha.org/) works with national programmes to provide free health services, including TB diagnostics and treatment, in India's urban slums and poor rural areas, where health care access is typically limited.^12^,^13^ Their approach includes community engagement, patient empowerment and biometric technology. During the study period, the organisation operated in seven Indian states (Figure 1), running TB centres in collaboration with local health care practitioners and the RNTCP.

We used data for patients diagnosed based on symptoms and sputum smears through Operation ASHA between April 2012 and September 2014. Following diagnosis, all TB patients initiate directly observed treatment (DOT), delivered by trained community members. Patients were included if they had pulmonary TB with an initial sputum smear result or extra-pulmonary TB, and were not multidrug-resistant (MDR, defined as TB that is resistant to both isoniazid [H] and rifampicin [R]) at presentation.

At each DOT visit, the provider records drug administration using biometric software (including fingerprint identification of patient and provider) on a tablet computer.^14^ Demographic and clinical information is entered at enrolment, including age, sex, initial sputum smear status, site of disease (pulmonary or extra-pulmonary; patients with both pulmonary and extra-pulmonary TB are classified as having pulmonary disease), and patient category (Category I, no previous anti-tuberculosis treatment or treatment for <1 month, or Category II, previous treatment for ⩾1 month).

### Treatment regimens

**FIGURE 2:**
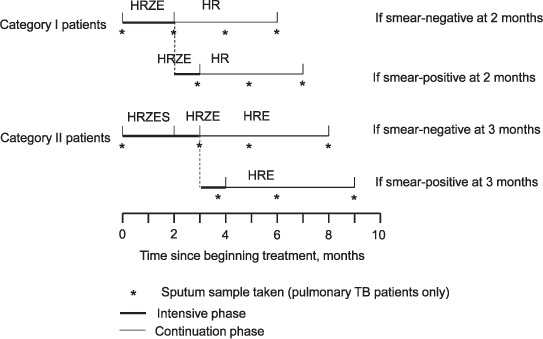
Treatment regimens used in Operation ASHA as recommended by the Indian national guidelines. H = isoniazid; R = rifampicin; Z = pyrazinamide; E = ethambutol; S = streptomycin; TB = tuberculosis.

Following India's national guidelines, treatment consists of an intensive phase including supervision of all doses, followed by a continuation phase with every third dose supervised.^15^ Treatment comprises three doses/week throughout and lasts 6–9 months, depending upon the patient category, and, for pulmonary TB patients, sputum smear status after the intensive phase (Figure 2). The intensive phase comprises 2 months of HR, pyrazinamide (Z) and ethambutol (E), plus injected streptomycin (S) for Category II patients, for whom the intensive phase also includes an additional month of HRZE only. At the end of the intensive phase, patients provide a sputum sample; if positive, treatment is continued for one further month. After this time, or at the time of the sputum sample if negative, patients move to the continuation phase: 4 months of HR for Category I patients or 5 months of HRE for Category II patients. Additional sputum samples are taken 2 months into the continuation phase and at treatment completion; patients with repeated positive results are evaluated and treated for MDR-TB if appropriate. Any missed doses are added to the end of treatment: all patients who complete treatment will have taken the full prescribed number of doses.

### Treatment outcomes

Treatment outcomes are defined in Table 1. In our primary analysis, outcomes were dichotomised into favourable (cured or treatment completed) and unfavourable/neutral (treatment failure, LTFU, death, switch to MDR-TB treatment or transferred out—hereafter described as unfavourable), consistent with WHO measures of treatment success.^3^

**TABLE 1:**
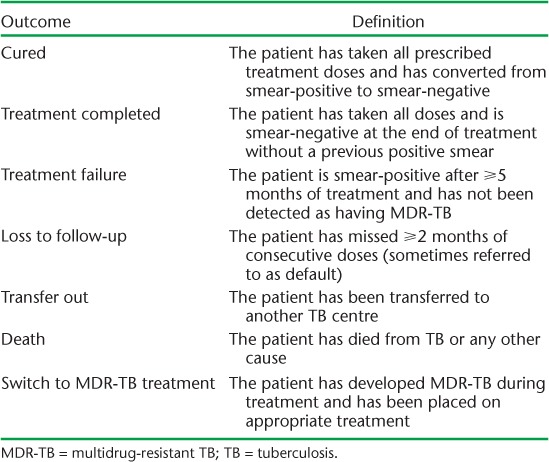
Definitions of treatment outcomes

### Statistical analysis

Data were initially summarised descriptively. The following explanatory variables were then assessed for associations with the binary treatment outcome using univariate mixed effects logistic regression to estimate odds ratios (ORs) and 95% confidence intervals (CIs), accounting for clustering by treatment centre and state: age group (⩽15, 16–24, 25–44 and ⩾45 years), sex, treatment category, urban/rural location of treatment centre and disease type. The latter combined site of disease and initial sputum smear status, and was categorised as pulmonary smear-negative, pulmonary low smear-positive (initial smear result scanty or 1+), pulmonary high smear-positive (2+ or 3+) or extra-pulmonary. Urban/rural location was classified following definitions used by the Census of India.^16^ Age group was treated as a linear rather than categorical variable after preliminary analysis found this produced a better-fitting model. A priori, we considered all of these factors as likely to be associated with treatment outcomes, so all were included in a multivariable model. *P* values were derived using likelihood ratio tests.

We conducted three sensitivity analyses. First, patients who were transferred out (final outcomes unknown) were excluded. Second, because repeat episodes for the same patient cannot be identified in the data set, Category II patients were excluded. Third, we restricted analysis to extra-pulmonary and smear-negative pulmonary patients to investigate risk factors for not completing treatment (the operational outcome definitions preclude patients with either extra-pulmonary or smear-negative pulmonary TB from being classified as cured).

Analyses were conducted in Stata v. 14 (StataCorp, College Station, TX, USA); maps were drawn using the user-written spmap command^17^ and shapefiles downloaded from the Global Administrative Areas database.^18^

### Ethical approval

Ethical approval was not sought, as data were collected during routine care and anonymised before analysis. Operation ASHA has permission from the RNTCP (New Delhi, India) to use anonymised data for research and public health purposes without informed consent.

## RESULTS

Between April 2012 and September 2014, 8415 patients with pulmonary disease and a sputum smear result at the beginning of treatment or with extra-pulmonary disease were enrolled in anti-tuberculosis treatment under Operation ASHA (Table 2). Patients were treated at 135 centres in seven states (range 1–453 patients per centre) (Figure 1).

**TABLE 2:**
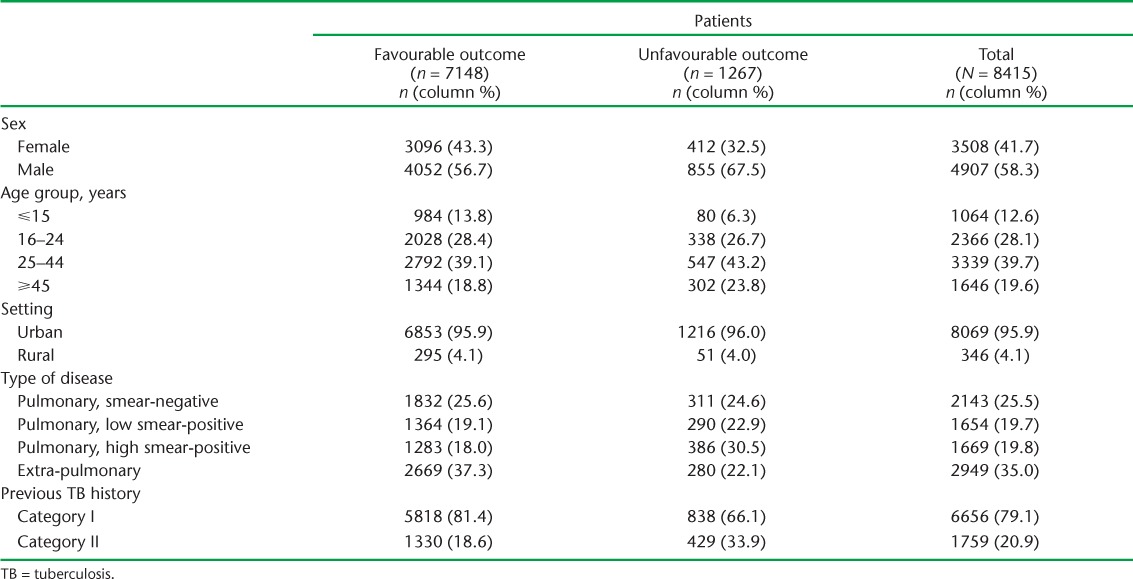
Demographic and clinical characteristics of TB patients enrolled in treatment through Operation ASHA and having initial smear results or extra-pulmonary TB, April 2012–September 2014

The majority of the patients (58.3%) were male; the highest percentage (39.7%) were aged 25–44 years (Table 2). Almost all (95.9%) attended urban treatment centres. At enrolment, 35.0% had extra-pulmonary TB, 25.5% had smear-negative pulmonary disease, 19.7% had a low positive smear result and 19.8% had a high positive result. Overall, 20.9% of patients had previously received anti-tuberculosis treatment, i.e., were Category II.

There were 7148 (84.9%) patients who had a favourable outcome (2588 [36.2%]) cured, 4560 [63.8%] treatment completed). Of 1267 patients with an unfavourable outcome, 82 (6.5%) experienced treatment failure, 381 (30.1%) were lost to follow-up, 281 (22.2%) died, 360 (28.4%) transferred out and 163 (12.9%) switched to MDR-TB treatment.

On univariate analysis, unfavourable outcomes were more common among males, in older age groups and in Category II vs. Category I patients (Table 3). Compared to pulmonary smear-negative patients, low and high smear-positive patients were increasingly likely to have an unfavourable outcome, while extra-pulmonary cases appeared less likely to have an unfavourable outcome. There was no evidence that outcomes differed between urban and rural settings.

**TABLE 3:**
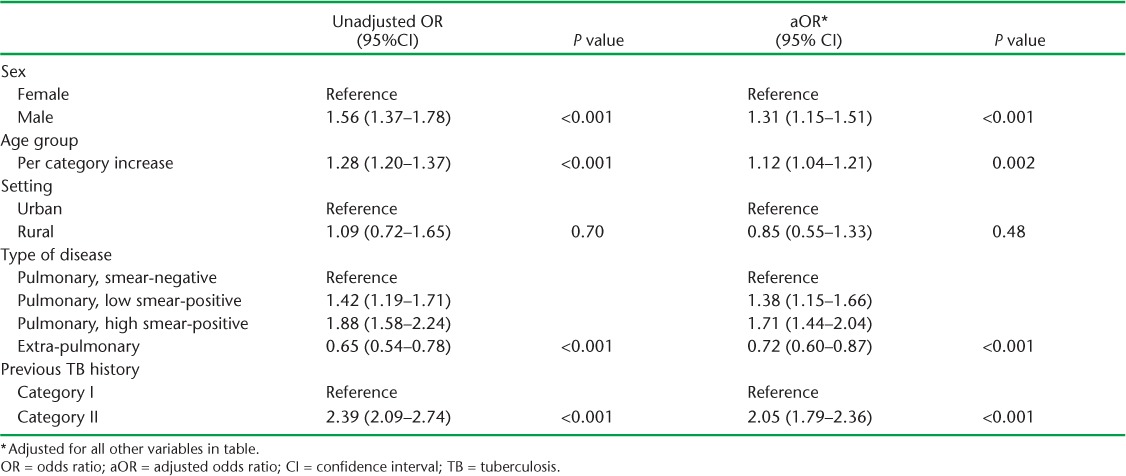
Unadjusted and adjusted OR for the association between explanatory variables and unfavourable treatment outcomes for all patients. All estimates are adjusted for clustering by treatment centre and state

On multivariable analysis, these associations remained, but were somewhat attenuated (Table 3). Males were more likely than females to have unfavourable outcomes (OR 1.31, 95%CI 1.15–1.51, *P* < 0.001). Age remained associated with increasing odds of an unfavourable outcome (OR 1.12, 95%CI 1.04–1.21, *P* = 0.002), as did increasing degree of sputum smear positivity (OR 1.38, 95%CI 1.15–1.66 and OR 1.71, 95%CI 1.44–2.04 for low and high positive pulmonary TB patients vs. smear-negative). Extra-pulmonary disease was associated with lower odds (OR 0.72, 95%CI 0.60–0.87). Category II patients had approximately twice the odds of having an unfavourable outcome as Category I patients (OR 2.05, 95%CI 1.79–2.36, *P* < 0.001).

Sensitivity analysis, excluding the 360 patients who were transferred out or the 1759 Category II patients, produced similar results (Table 4). Results based on the 5092 patients with pulmonary smear-negative (*n* = 2143) or extra-pulmonary (*n* = 2949) disease were again similar (Table 4), although the OR for treatment category was reduced (OR 1.50, 95%CI 1.20–1.86, *P* < 0.001).

**TABLE 4:**
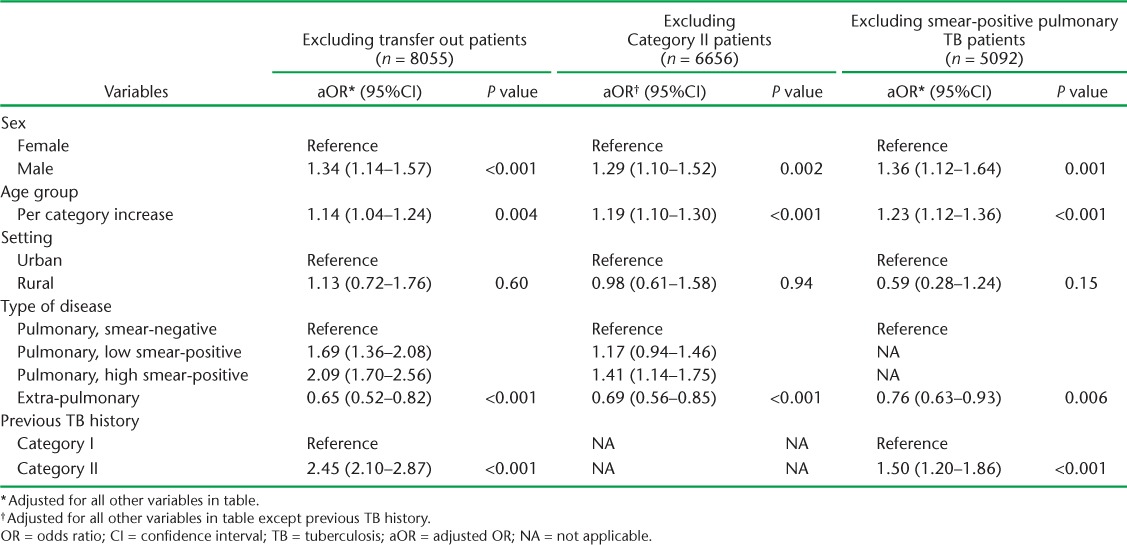
Adjusted ORs and 95%CIs for the association between explanatory variables and unfavourable treatment outcomes obtained in sensitivity analyses which excluded 1) patients with a recorded outcome of transfer out, 2) patients with a recorded history of previous anti-tuberculosis treatment (Category II patients), or 3) patients with smear-positive pulmonary disease. All estimates are adjusted for clustering by treatment centre and state

## DISCUSSION

In this cohort from a disadvantaged population of TB patients in India, risk factors for unfavourable treatment outcomes were increasing age, male sex, history of TB treatment and having pulmonary, and particularly smear-positive, disease. Overall, Operation ASHA successfully treated 84.9% of the patients in our analysis. This is comparable to national data reported from India (treatment success rate of 88% among new and relapse cases registered in 2013,^19^ 74% for 2014^1^), and approaches the 90% target set for 2025 in the WHO's End TB strategy,^20^ despite the fact that Operation ASHA works with highly vulnerable patients. Previous studies in India have reported similar treatment success rates, e.g., 76.7% in patients positive for the human immunodeficiency virus (HIV) and 93.5% in HIV-negative patients,^21^ 93% with community DOT and 75% with institutional DOT,^22^ and 74.3% in HIV-positive and 79.9% in HIV-negative patients.^23^ While we are not aware of any estimates for drug-susceptible TB specifically for highly disadvantaged populations, 48% of 23 HIV-positive MDR-TB patients in a Mumbai slum had favourable outcomes.^24^

The age and sex distribution of TB patients treated by Operation ASHA differed from that of TB patients in India nationally: in the national data, 9% of patients are aged <15 years and 35% are female,^1^ compared to respectively 12.6% and 41.7% in our data set. A lower percentage of Operation ASHA patients have pulmonary disease (65.0%) compared to national data (82%).^1^ These differences may reflect Operation ASHA's aim to reach patients who otherwise may never receive care.

The relationships we observed between treatment outcome and age, sex and previous treatment have been previously reported, in India and elsewhere.^10^,^11^,^25–27^ In a rural area of South India in 1999–2000, the odds of LTFU were higher among men than women (adjusted OR [aOR] 3.4, 95%CI 1.5–8.2), in patients aged ⩾45 years compared to younger patients (aOR 1.6, 95%CI 1.0–2.6), and in previously treated patients compared to those with no previous treatment (aOR 2.8, 95%CI 1.6–4.9).^11^ In contrast with our results, that study found no association between site of disease and LTFU, although only univariate results were presented and the point estimate suggested higher rates of LTFU among extra-pulmonary patients (OR 2.8, 95%CI 0.99–11.1).^11^ Our comparison of urban and rural settings is limited by the small number of patients in rural areas.

Although the data were very complete for the included covariates, we lacked information on several relevant exposures. Importantly, HIV status is not well reported in the data set. In Indian national data, 4% of TB patients with known HIV status are HIV-positive;^1^ an HIV prevalence of 1.2% was reported for men living in slums in Chennai in 2001–2002.^28^ We also lacked data on smoking. Among patients attending a designated microscopy centre in South India, 41.5% of presumptive TB patients and 80% of smear-positive pulmonary TB patients reported tobacco use in the previous month.^29^ Smoking may therefore be common in the study population, increasing the risk of poor outcomes.^11^,^30–32^ Although we had no data on SEP, the populations with whom Operation ASHA works are extremely disadvantaged even in comparison to other TB patients in India. The great majority of Operation ASHA's patients (96%) live in urban slums. Slum residents have previously been identified as a high-risk group for TB in India (aOR 1.6, 95%CI 1.00–2.45 for culture-positive pulmonary TB),^8^ although disease is not confined to this group; the prevalence was 0.4% in slum dwellers and 0.2% in other residents of urban Chennai.^8^

Previous studies of pulmonary TB outcomes according to smear status have produced varying results.^33^ This has been suggested to be related to HIV prevalence, especially advanced HIV disease, which is associated with low bacillary burden in the sputum.^34^ While extra-pulmonary TB has been associated with longer treatment delays,^35^ HIV infection^36^ and worse outcomes than pulmonary TB,^26^ higher treatment success among extra-pulmonary patients has also been reported.^37^ This depends partly on the site of extra-pulmonary disease; for example, meningeal and disseminated TB have higher mortality rates than lymphatic TB.^38^ As patients with solely extra-pulmonary disease will not have a positive sputum smear at enrolment, they cannot be classified as ‘cured’, which requires smear conversion. We therefore conducted a sensitivity analysis restricted to extra-pulmonary and initially smear-negative pulmonary cases; the association between site of disease and outcome persisted. This could result from residual confounding by measured or unmeasured factors, such as treatment adherence, or because patients with both pulmonary and extra-pulmonary disease were classified as pulmonary cases. A univariate analysis of data from the RNTCP on TB-HIV patients, however, reported lower treatment success amongst pulmonary cases (OR 0.58, 95%CI 0.40–0.83),^37^ consistent with our results.

Our analysis does not include patients with MDR-TB. Risk factors for poor outcomes of MDR-TB, particularly in disadvantaged populations, should be further investigated, as rates of treatment failure are higher in MDR-TB than in drug-susceptible TB.^11^,^25^

Current WHO treatment guidelines recommend daily dosing throughout treatment in both the intensive and continuation phases.^39^ If this is not feasible, recommended alternatives are daily dosing during the intensive phase and three times per week during the continuation phase, all directly observed; or, for patients not living with HIV and not in a setting with high HIV prevalence, directly observed doses three times weekly throughout the intensive and continuation phases.^39^ The treatment regimen used by Operation ASHA, and recommended by the RNTCP, maintains the WHO-recommended dosing frequency, but with direct observation of only every third dose during the continuation phase. Further clinical follow-up data on relapses are needed to better assess the adequacy of this regimen in this population.

The percentage of patients who are successfully treated depends partly on mortality rates from causes other than TB, which may be high in this population and might reduce the treatment success rate. Without cause-specific mortality data, this is difficult to address. Our approach is consistent with the WHO's method of assessing treatment success. Deaths from other causes undoubtedly contribute to the increased risk of unfavourable outcome in older age groups, but other factors, including delayed presentation^35^ and difficulties in diagnosis and treatment^40^ in the elderly, may also be relevant.

## CONCLUSIONS

Operation ASHA appears to be achieving treatment success rates comparable to those reported through national surveillance^1^,^3^ and epidemiological studies conducted in India.^21–23^ However, we have not formally evaluated the programme, including its biometric technology and electronic data recording components, and our analysis does not contain a comparison group, which would be necessary to assess the effectiveness and cost-effectiveness of the service. Reaching vulnerable populations such as those described in this study will be essential if the goals of the End TB strategy^20^ are to be achieved.
